# Enzymatic Redox Properties and Cytotoxicity of Irreversible Nitroaromatic Thioredoxin Reductase Inhibitors in Mammalian Cells

**DOI:** 10.3390/ijms241512460

**Published:** 2023-08-05

**Authors:** Aušra Nemeikaitė-Čėnienė, Lina Misevičienė, Audronė Marozienė, Violeta Jonušienė, Narimantas Čėnas

**Affiliations:** 1 Department of Immunology of State Research Institute Center for Innovative Medicine, Santariškiu˛ St. 5, LT-08406 Vilnius, Lithuania; ausra.ceniene@imcentras.lt; 2 Department of Xenobiotics Biochemistry, Institute of Biochemistry of Vilnius University, Sauletekio 7, LT-10257 Vilnius, Lithuania; lina.miseviciene@bchi.vu.lt (L.M.); audrone.maroziene@bchi.vu.lt (A.M.); 3 Department of Biochemistry and Molecular Biology, Institute of Biosciences of Vilnius University, Sauletekio 7, LT-10257 Vilnius, Lithuania; violeta.jonusiene@gf.vu.lt

**Keywords:** nitroaromatic compounds, cytotoxicity, oxidative stress, thioredoxin reductase, inhibition of

## Abstract

NADPH:thioredoxin reductase (TrxR) is considered a potential target for anticancer agents. Several nitroheterocyclic sulfones, such as Stattic and Tri-1, irreversibly inhibit TrxR, which presumably accounts for their antitumor activity. However, it is necessary to distinguish the roles of enzymatic redox cycling, an inherent property of nitroaromatics (ArNO_2_), and the inhibition of TrxR in their cytotoxicity. In this study, we calculated the previously unavailable values of single-electron reduction potentials of known inhibitors of TrxR (Stattic, Tri-1, and 1-chloro-2,4-dinitrobenzene (CDNB)) and inhibitors identified (nitrofuran NSC697923 and nitrobenzene BTB06584). These calculations were according to the rates of their enzymatic single-electron reduction (PMID: 34098820). This enabled us to compare their cytotoxicity with that of model redox cycling ArNO_2_. In MH22a and HCT-116 cells, Tri-1, Stattic, CDNB, and NSC697023 possessed at least 10-fold greater cytotoxicity than can be expected from their redox cycling activity. This may be related to TrxR inhibition. The absence of enhanced cytotoxicity in BTB06548 may be attributed to its instability. Another known inhibitor of TrxR, tetryl, also did not possess enhanced cytotoxicity, probably because of its detoxification by DT-diaphorase (NQO1). Apart from the reactions with NQO1, the additional mechanisms influencing the cytotoxicity of the examined inhibitors of TrxR are their reactions with cytochromes P-450. Furthermore, some inhibitors, such as Stattic and NSC697923, may also inhibit glutathione reductase. We suggest that these data may be instrumental in the search for TrxR inhibitors with enhanced cytotoxic/anticancer activity.

## 1. Introduction

Nitroaromatic compounds (ArNO_2_) are widely used as antiparasitic, antibacterial, antitumor, and radiosensitizer agents ([1,2,3,4,5] and references therein). Frequently, these activities of ArNO_2_ are exerted through their single-electron enzymatic reduction with the formation of radical anions (ArNO_2_^−^·) or through a two/four-electron reduction with the net formation of hydroxylamines (ArNHOH). The former reaction, carried out by flavoenzyme electron-transferases, e.g., NADPH:cytochrome P-450 reductase (P-450R) or ferredoxin-type FeS proteins [6,7,8], initiates the redox cycling of ArNO_2_^−^· and causes oxidative stress-type cytotoxicity. The latter reaction, catalyzed mainly by NAD(P)H:quinone oxidoreductase (DT-diaphorase, NQO1) in mammalian cells, leads to the alkylation of DNA and other cellular nucleophiles [9,10]. Another relevant type of redox transformation of ArNO_2_ is its denitration catalyzed by cytochromes P-450 with the formation of corresponding hydroxy derivatives [11,12]. The reaction intermediates, epoxides, react with thiol groups. Other modes of cytotoxic and therapeutic action of ArNO_2_, in particular those associated with inhibiting certain enzymes, are discussed in recent reviews [5,13].

In this context, NADPH:thioredoxin reductase (TrxR) is considered a potential target for anticancer agents because it is overexpressed in numerous cancer lines [14,15,16,17]. Mammalian TrxRs have three cofactors in their active center: FAD, catalytic disulfide, and selenylsulfide. Selenylsulfide is located at the *C*-end of the protein. The physiological substrates of TrxRs are 10–12 kD disulfide proteins called thioredoxins (Trxs), which perform antioxidant and other numerous physiological functions [18]. During catalysis, the redox equivalents are transferred in the following sequence: NADPH → FAD → catalytic disulfide → selenylsulfide. Subsequently, selenylsulfide reduces Trx or the artificial oxidant 5,5′-dithiobis(2-nitrobenzoic acid) (DTNB). The maximal reduction rate of selenylsulfide is close to that of Trx reduction [19].

**Figure 1:**
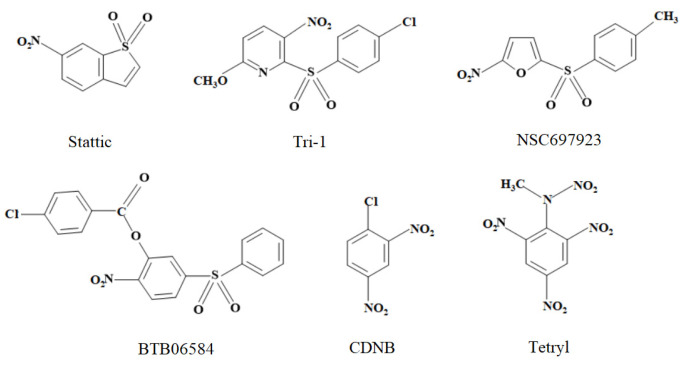
The structures of nitroaromatic compounds studied in this work.

Typically, TrxRs slowly reduce various groups of ArNO_2_ in a one- or two-electron way, with FAD and/or catalytic selenylsulfide involved in the reactions [20,21,22]. On the other hand, several nitroheterocyclic compounds containing a sulfone group, such as Stattic and Tri-1 (Figure 1) or their derivatives, rapidly and irreversibly inhibit TrxR, which presumably accounts for their antitumor activity [22,23]. The therapeutic potential of Tri-1 and Stattic has been demonstrated in several non-tumor cell lines and human tumor xenografts in mice [23,24].

However, to the best of our knowledge, redox activity and redox cycling are inherent properties of nitroaromatics, which have not been studied for the above compounds. Therefore, it is important to determine whether and to what extent TrxR inactivation increases their cytotoxicity compared to their non-specific cytotoxicity, which is mainly caused by redox cycling.

In this work, we examined the kinetics of the single-electron enzymatic reduction in Stattic and Tri-1. This allowed us to quantitatively characterize their redox cycling properties. For comparison, we studied the representatives of other groups of nitroaromatic sulfones: an inhibitor of ubiquitin-conjugating enzymes, nitrofuran NSC697923 [25,26], and an inhibitor of ATP-ase, nitrobenzene derivative BTB06584 [27]. Additionally, we investigated two well-known irreversible nitroaromatic inhibitors of TrxR, 1-chloro-2,4-dinitrobenzene (CDNB) and tetryl [20,21], which do not have a sulfonic group (Figure 1). Further enzymatic and mammalian cell cytotoxicity studies enabled us to conclude that the cytotoxicity of Stattic, Tri-1, NSC697923, and CDNB significantly exceeds the limits predicted by their redox cycling activity. This enhanced cytotoxicity may be attributed to the inhibition of TrxR. However, some additional cytotoxicity mechanisms of these compounds may also be considered.

## 2. Results

### 2.1. Enzymatic Redox Properties of Examined Nitroaromatic Compounds

It is commonly accepted that if the main cytotoxicity factor of ArNO_2_ is the formation rate of ArNO_2_^−^· and their redox cycling, their cytotoxicity may be described by a general quantitative structure-activity relationship:

log cL_50_ = *a* − *b E*^1^_7_ − *c* log *P* (log *D*), (1)

where *E*^1^_7_ is the midpoint redox potential of the ArNO_2_/ArNO_2_^−^· couple (single-electron reduction potential at pH 7.0), cL_50_ is the compound concentration causing 50% cell death or an analogous quantitative parameter, log *P* is the octanol/water partition coefficient, and log *D* is the octanol/water distribution coefficient at pH 7.0 [28,29,30]. These dependencies mirror the linear log (rate constant) vs. *E*^1^_7_ relationships in a single-electron reduction of ArNO_2_ by P-450R or other single-electron transferring flavoenzymes [6,30]. One may suppose that exceeding the limits predicted by the redox cycling activity may point to additional mechanisms of cytotoxicity or therapeutic action of ArNO_2_. These mechanisms might include direct or bioreductively activated alkylation, DNA intercalation, or the inhibition of particular enzymes [13].

We evaluated the unavailable *E*^1^_7_ values of tested nitroaromatics (Figure 1), as previously, by studying their single-electron reduction kinetics by P-450R and the Fe_2_S_2_ redox protein adrenodoxin (ADX) [31]. Flavoenzyme NADPH:adrenodoxin reductase (ADR) reduces ArNO_2_ in a single-electron way, but the process is slow due to the inhibition of the reaction by excess NADPH. ADX eliminates the inhibition by NADPH and confers an additional reduction pathway via reduced ADX [32]. In both enzymatic systems, the log of the steady-state bimolecular reduction rate constants (*k*_cat_/*K*_m_) of ArNO_2_ linearly increases with *E*^1^_7_ [31]. This may be attributed to an “outer-sphere” electron transfer mechanism, where the reactivity of homologous compounds is insignificantly influenced by their structural peculiarities [33]. Thus, the unavailable *E*^1^_7_ values of ArNO_2_ may be predicted from their log *k*_cat_/*K*_m_, whereas their geometric average obtained in several systems (log *k*_cat_/*K*_m_(avge)) improves the prediction accuracy [31].

In P-450R-catalyzed reactions, the reaction rates were proportional to the concentration of NSC697923 and BTB06548 up to the limits of their solubility, 70  µM and 200 µM for other compounds. The reduction rates by ADR/ADX exhibited parabolic dependence on their concentration, giving a *k*_cat_ of 3.5–4.0 s^−1^, which was close to 50% of the ADX-mediated cytochrome *c* reduction rate. The *k*_cat_/*K*_m_ values of ArNO_2_ reduction are given in Table 1.

The previously determined log *k*_cat_/*K*_m_(avge) of model ArNO_2_ in P-450R- and ADR/ADX-catalyzed reactions (Appendix A, Table A1) well correlate with their *E*^1^_7_ values (Appendix A, Equation (A1)). The calculated *E*^1^_7_ values of ArNO_2_ (*E*^1^_7(calc.)_) obtained using this equation are given in Table 1. These values could be realistic because the *E*^1^_7(calc.)_ of NSC697923 is similar to those of other nitrofurans, and the *E*^1^_7(calc.)_ value of CDNB is higher than that of *m*-dinitrobenzene due to the electron-accepting character of the chlorine substituent (Appendix A, Table A1). In turn, the *E*^1^_7(calc.)_ value of Tri-1 (Table 1) is significantly lower than that of *p*-nitropyridine, −0.19 V [34]. This is because of its *m*-nitropyridine structure and the electron-donating character of the methoxy group.

Flavoenzyme NAD(P)H:quinone oxidoreductase (NQO1) reduces quinones in a two-electron way, with *k*_cat_ in certain cases being above 1000 s^−1^ [35]. However, the reduction in ArNO_2_ with the formation of ArNHOH is 10^2^–10^5^ times slower than that of quinones [36] due to insufficiently defined reasons. The rate constants of NQO1-catalyzed reduction of nitroaromatics were calculated from the rate of NADPH oxidation monitored at 340 nm. To account for possible changes in ArNO_2_ absorbance at 340 nm occurring during reduction, control experiments were conducted using the NADPH regeneration system, glucose-6-phosphate, and glucose-6-phosphate dehydrogenase. However, these changes amounted to no more than 10% of the total absorbance changes at 340 nm. One may note that the reactivity of the examined compounds is low (Table 1). For comparison, one of the fastest nitroaromatic substrates of NQO1, tetryl, is characterized by *k*_cat_ = 73 s^−1^ and *k*_cat_/*K*_m_ = 2.6 × 10^5^ M^−1^s^−1^ [36].

Another relevant problem is the possible action of tested nitroaromatics as ‘subversive substrates’ for TrxRs, where their reduction into ArNO_2_^−^· may lead to toxic effects [21]. We also investigated the reduction in these compounds by the structurally and functionally related antioxidant flavoenzyme human glutathione reductase (HGR) [19]. Among ArNO_2_, tetryl was the most efficient oxidant of mammalian TrxRs, with a *k*_cat_ of 1.8 s^−1^ (rat cytosolic enzyme (TrxR1) [21]), 2.9 s^−1^ (human TrxR1), and 0.7 s^−1^ (human mitochondrial enzyme (TrxR2)) [37], which is about 9% of the *k*_cat_ of DTNB reduction and of HGR, with *k*_cat_ ≥ 5 s^−1^ and *k*_cat_/*K*_m_ = 2.0 × 10^3^ M^−1^s^−1^ [38]. Purified human TrxR2 and commercially available rat TrxR1 preparations used in this study possessed NADPH:oxidase activities of 0.02 s^−1^ and 0.01 µmol NADPH oxidized ·min^−1^ ·mg protein, respectively. The presence of 100 µM Stattic, Tri-1, or CDNB, or 70 μM BTB06584 or NSC697923 increased the NADPH:oxidase activity of TrxRs by 2–2.5 times, while 100 μM tetryl increased it by 12–14 times. In reactions with HGR, the same concentrations of Stattic, Tri-1, CDNB, or BTB06584 increased its NADPH:oxidase activity from 0.04 s^−1^ only to 0.08–0.09 s^−1^. The activity of NSC697923 was higher, with *k*_cat_ ≥ 0.35 s^−1^ and *k*_ca_t/*K*_m_ = 3.0 ± 0.4 × 10^3^ M^−1^s^−1^. The reduction rate of 50 µM cytochrome *c* added into the reaction mixture was 60% of the NADPH oxidation rate, i.e., the single-electron flux during NSC697923 reduction was 30% [21,38]. Thus, our data show that the examined compounds are very slow ‘subversive substrates’ for TrxRs and HGR, with several times lower reactivity than tetryl.

### 2.2. Reactions of Nitroaromatic Compounds with Reduced Glutathione

Nitroaromatic sulfones react with reduced glutathione (GSH) (Equation (2)) [39] and other cellular thiols:

O_2_N-Ar_1_-SO_2_-Ar_2_ + GSH → O_2_N-Ar_1_-SG + HO_2_S-Ar_2_. (2)

They inactivate reduced TrxR in a similar reaction, reacting predominantly with catalytic selenol [23]. On the other hand, the reaction with GSH may be considered a pathway to their detoxification. The pseudo-first-order reaction rate constants of the examined nitroaromatic sulfones (Figure 1) were directly proportional to GSH concentration (2.0–10.0 mM). The calculated second-order rate constants decreased in the order NSC697923 (7.2 ± 0.3 M^−1^s^−1^), Tri-1 (4.8 ± 0.3 M^−1^s^−1^), and Stattic (1.1 ± 0.1 M^−1^s^−1^). BTB06584 was found to be relatively unstable, with its λ_max_ gradually shifting from 280 nm to 316 nm with t_1/2_ ~ 1.5 h at pH 7.0 and 37 °C. Therefore, the rate constant of its reaction with GSH was estimated to be only approximately ~0.5 M^−1^s^−1^ at 25 °C. For comparison, the rate constants of the reaction of GSH with tetryl and CDNB under the same conditions are 1.2 M^−1^s^−1^ and 0.03 M^−1^s^−1^, respectively [20,21].

### 2.3. Cytotoxicity of Nitroaromatic Compounds

By studying a series of ArNO_2_ without alkylating or bioreductively activated alkylating groups, we found that their concentration causing 50% MH22a cell death (cL_50_) or concentration causing 50% growth inhibition of HCT-116 cells (GI_50_) is described by a two-parameter regression similar to that presented in Equation (1) [31,40]. After supplementing these dependencies with new data, their final expression is given in Equations (A2) and (A3) of Appendix B. The cL_50_ and GI_50_ of nitroaromatic sulfones and 1-chloro-2,4-dinitrobenzene determined in this work are presented in Table 2.

**Figure 2:**
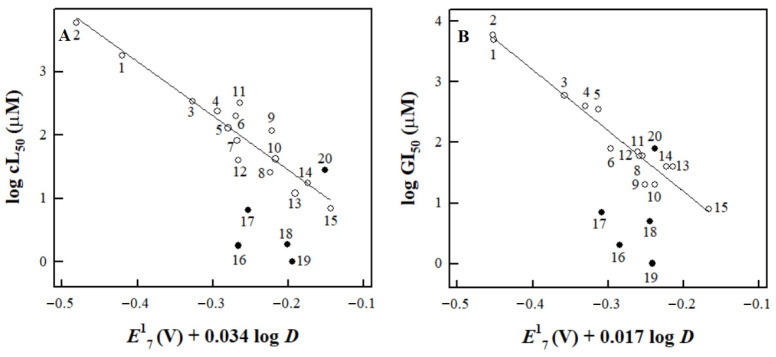
The dependence of the cL_50_ of nitroaromatic compounds against MH22a cells (**A**) and their GI_50_ against HCT-116 cells (**B**) on their *E*^1^_7_ and log *D* as described by Equations (A2) and (A3) (Appendix B). The correlation lines are drawn according to these equations for compounds 1–15 (Table A2, Appendix B). The compounds examined in this study are tetryl (15), Stattic (16), Tri-1 (17), CDNB (18), NSC697923 (19), and BTB06584 (20).

The data in Figure 2A,B show that Tri-1, Stattic, NSC697923, and 1-chloro-2,4-dinitrobenzene exhibit significantly greater activity than would be expected from their *E*^1^_7(calc.)_ and log *D*. However, the increase in cytotoxicity is not characteristic of BTB06584, nor of another irreversible inhibitor of TrxR, tetryl (Figure 2A,B).

Table 2 also shows their expected cL_50_ and GI_50_ values calculated from Equations (A2) and (A3) (Appendix B). One may note that the GI_50_ of Tri-1 in HCT-116 cells (Table 2) is comparable to the value previously determined for the same cells at 48 h, 2.9 μM [23].

The cytotoxicity of nitroaromatics in MH22a cells was decreased by desferrioxamine and the antioxidant *N*,*N*’-diphenyl-*p*-phenylene diamine (DPPD) (Table 3). Concerning the other enzymatic mechanisms, we examined the effects of an inhibitor of NQO1, dicoumarol, and several inhibitors of cytochromes P-450 on the cytotoxicity of ArNO_2_ [31,40] (Table 3). Dicoumarol decreased the cytotoxicity of the compounds except for tetryl, and cytochrome P-450 inhibitors did so in most cases, except for the increase in toxicity of Stattic in the presence of α-naphthoflavone and isoniazid and the increase in toxicity of tetryl in the presence of miconazole (Table 3). Because of the instability of BTB06584 at pH 7.0 and 37 °C, further cytotoxicity studies were not performed.

### 2.4. Inhibition of Thioredoxin Reductase and Glutathione Reductase by Nitroaromatic Compounds

In most previous studies, the efficiency of TrxR inhibition by ArNO_2_ was evaluated by the decrease in the activity of the enzyme after incubation of its NADPH-reduced form with the compound for a certain time [20,21,22,23]. However, this does not allow for a separate assessment of their reversible inhibition, which is independent of time, and irreversible inhibition, which usually occurs using covalent modification and is time-dependent. In our initial experiments, we used the lysates of MH22a and HCT-116 cells, in which the activity of TrxR was 56.2 ± 3.2 and 42.9 ± 3.0 nmol DTNB reduced ·min^−1^ ·mg protein, respectively. The activity of GR was 302 ± 18 and 100 ± 7.0 nmol NADPH oxidized ·min^−1^ ·mg protein, respectively.

**Figure 3:**
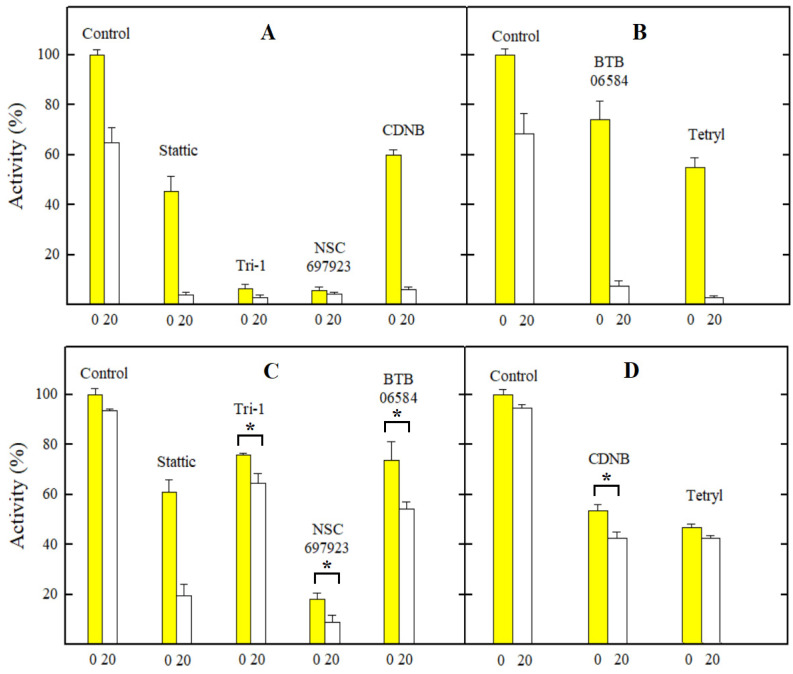
The changes in the activity of TrxR (**A**,**B**) and GR (**C**,**D**) in the lysates of MH22a cells (**A**,**C**) and HCT-116 cells (**B**,**D**) under the action of the examined nitroaromatic compounds. The numbers on the abscissas indicate the minutes of lysate incubation time with NADPH in the presence or absence of the compound. The asterisks (*) indicate a statistically significant difference with *p* ≤ 0.05. In all other cases except the inactivation of GR in MH22a cell lysates (**C**) and the action of tetryl on GR in HCT-116 cell lysates (**D**), the differences between t = 0 and t = 20 min are characterized by *p* = 0.01–0.001.

First, we investigated the effects of ArNO_2_ on the enzyme activity by introducing them immediately into the reaction mixture (t = 0). In the second case, we incubated the lysate with NADPH and tested the compound for 20 min, which was close to the conditions of previous studies [20,21,22,23]. The concentration of compounds used was 10 µM, which was several times higher than the cL_50_ or GI_50_ of the most toxic compounds, CDNB, Tri-1, and NSC697923 (Table 2). It can be seen that at this compound concentration, the residual activity of TrxR in the presence of Tri-1 and NSC697923 is very small, both at t = 0 and t = 20 min (Figure 3A). However, a more pronounced time-dependent inhibition of GR was observed only under the action of Stattic, while in the cases of CDNB, Tri-1, and SN697923, it was less pronounced and almost absent in the case of tetryl (Figure 3B). It should be noted that experiments with all tested compounds in both cell lysates gave very similar results when comparing the relative inhibition of the reaction rate. Therefore, in Figure 3, we present only one case of TrxR and HGR inhibition by each compound.

Next, we investigated the inhibitory effects of ArNO_2_ on the preparation of rat TrxR1 and purified HGR (Table 4). This enabled us to quantitatively separate the effects of reversible and irreversible inhibition. Our data demonstrate that NSC697923 and Tri-1 reversibly inhibit TrxR1 at micromolar concentrations and further rapidly inactivate it (Table 4). On the other hand, while Stattic, CDNB, and tetryl reversibly inhibit TrxR1 at higher concentrations, they caused its rapid inactivation, with the activity of tetryl exceeding that of CDNB (Table 4). This is in line with the results of previous observations [21,23]. It should be noted that Stattic rapidly inactivates HGR, while Tri-1, tetryl, and CDNB are almost inactive (Table 4), which is again in line with the previous findings [21,24,38]. Because of the instability of BTB06584, further studies of its inhibitory properties were not carried out. Taken together, the effects of the studied nitroaromatics on the activity of isolated TrxR1 and HGR (Table 4) and their activity in the cell lysates (Figure 3) closely match each other.

## 3. Discussion

In this study, for the first time, we obtained reliable *E*^1^_7(calc.)_ values for several nitroaromatic compounds that were known or identified as irreversible inhibitors of TrxR (Figure 1, Table 1). This allowed us to quantitatively compare their cytotoxicity with that of model ArNO_2_. Their prooxidant cytotoxicity was demonstrated by the protective effects of desferrioxamine and DPPD (Table 3). Indeed, four of them, CDNB, NSC697923, Stattic, and Tri-1, were more cytotoxic in MH22a and HCT-116 cells than may be expected from their redox cycling activity and lipophilicity (Equations (A2) and (A3) (Appendix B), Table 2, Figure 2). Thus, it is possible to attribute this effect to their efficient irreversible inhibition of TrxR (Figure 3, Table 4). It can be noted that there is a certain parallelism between the rates of TrxR inactivation by these compounds (*k*_i_, Table 4) and their reactivity with GSH (Section 2.2). The data in Table 3 point to some additional mechanisms of action of the examined compounds: (i) Like other ArNO_2_, they react with cytochromes P-450 [11,12,31]. However, the mechanisms and roles of individual cytochromes P-450 remain to be elucidated. Furthermore, these reactions have different effects on their cytotoxicity. This complements the data on the observed transformation of Tri-1 in microsomes [23], and (ii) except for tetryl, these compounds are also activated by NQO1 (Table 3), most likely due to the formation of alkylating ArNHOH [9]. Moreover, no connection was observed between their activity as TrxR ‘subversive substrates’ and cytotoxicity. In our opinion, the role of these reactions in the cytotoxicity of the above compounds is negligible because the most active ‘subversive substrate’ of TrxR, tetryl, does not possess enhanced cytotoxicity. We have to mention separately that a novel efficient irreversible inhibitor of TrxR, NSC697923 (Figure 3, Table 4), was identified in this work. This compound also possessed the highest cytotoxicity among the examined compounds (Figure 2). This compound may have further application prospects due to its demonstrated antitumor and antiviral activity, including the inhibition of the growth of implanted human tumors without causing toxic effects in mice [26,41,42].

On the other hand, BTB06584 and tetryl efficiently inhibited TrxR in cell lysates in short-term experiments (Figure 3) but did not display enhanced cytotoxicity (Figure 2). This can be explained by the instability of BTB06584, which may affect the results of long-term cytotoxicity experiments. As for tetryl, the absence of its enhanced cytotoxicity has also been observed in both cancerous and normal cell lines [28,43]. It is important to note that although tetryl more rapidly than CDNB irreversibly inhibits isolated TrxR (Table 4), it was less effective than CDNB in inhibiting TrxR in the cells [21]. The available data on the TrxR inhibition in the cells by the compounds examined in this study are summarized in Table 5.

Therefore, it can be assumed that the inhibitory effect of tetryl becomes less pronounced in long-term cytotoxicity experiments. A possible reason for this phenomenon is that the enzymatic single-electron reduction and redox cycling of tetryl, especially its NOQ1-catalyzed single- and two-electron reduction, produce its less toxic metabolite, *N*-methylpicramide. This metabolite does not react with TrxR [36]. In favor of this argument is the fact that, unlike in the cases of other ArNO_2_, the NQO1 inhibitor dicoumarol increases but does not decrease the cytotoxicity of tetryl (Table 3). The analogous data were obtained in other cell lines [30].

There are a couple of other issues that need to be discussed regarding the inhibition of TrxR by both ArNO_2_ and other types of compounds, which may be taken as directions for further research. First, this is a characterization of the reversible inhibition of TrxR and its possible role in the cytotoxicity of inhibitors. Although most studies concerned the irreversible inhibition of enzymes [20,21,22,23], reversible inhibition of TrxR by ArNO_2_ was also demonstrated, which can be competitive or noncompetitive with respect to DTNB [21,45]. In the first case, this indicates that the inhibitor binds to the catalytic selenocysteine. However, the analysis of these data was complicated by the rapid inactivation of the enzyme due to its covalent modification by the inhibitor [21]. In the second case, this suggests that the inhibitor binds at a distinct site, most likely in the intersubunit domain. This domain is structurally close to the analogous HGR domain, according to crystallographic studies [46]. Our preliminary data obtained at saturating concentrations of NADPH and DTNB suggest that this type of inhibition may also be characteristic of TrxR, occurring even at micromolar concentrations of NSC697923 and Tri-1 (Table 4). The role of this phenomenon is almost unexplored, except for the data on the contribution of TrxR non-competitive inhibition by *N*-(4-chlorophenyl)-5-nitrofuran-2-carboxamide (LCS3) to its anticancer in vitro activity [45]. Analogously, it was found that the antiplasmodial in vitro activity of a series of ArNO_2_ was partly determined by their noncompetitive inhibition of the structurally related enzyme, *Plasmodium falciparum* GR [47]. Taken together, these data provide some new guidelines for the search for TrxR inhibitors with cytotoxic/anticancer activity, including studies of mutants of its active center residues [48].

The second issue is the concept of dual targeting of the glutathione and thioredoxin antioxidant systems because of their interconnection and compensatory nature, which has recently gained attention in cancer chemotherapy ([45,49] and references therein). It is suggested that the uncompetitive reversible inhibition of both TrxR and GR by nitrofuran LCS3 causes synergistic effects that enhance cell death [45]. In this aspect, one may note that the dual inhibition of TrxR and GR by Stattic is well documented [22,24] and supported by the results of the current study (Figure 3, Table 4). On the other hand, a newly identified compound, NSC697923, is more active than Stattic in this respect (Figure 3 and Table 4) and deserves more thorough studies.

## 4. Materials and Methods

### 4.1. Enzymes and Chemicals

Recombinant rat P-450R, bovine ADR, and ADX were prepared as described in [50]; their concentrations were determined according to ε_456_ = 21.4 mM^−1^·cm^−1^, ε_450_ = 11.0 mM^−1^·cm^−1^, and ε_414_ = 10.0 mM^−1^·cm^−1^, respectively. They were a generous gift from Dr. Alexey Yantsevich (Institute of Bioorganic Chemistry, BNAS, Minsk, Belarus). Recombinant HGR was prepared as previously described [51]; its concentration was determined according to ε_463_ = 11.3 mM^−1^·cm^−1^. It was a generous gift from Dr. Elisabeth Davioud-Charvet (Universite de Strasbourg, Strasbourg, France). Recombinant human mitochondrial thioredoxin reductase (TrxR2) was prepared as described in [52], and its concentration was determined according to ε_463_ = 11.3 mM^−1^·cm^−1^. It was a generous gift from Professor Elias Arner (Karolinska Institutet, Stockholm, Sweden). Tetryl was synthesized as described in [30]; it was a generous gift from Dr. Jonas Šarlauskas (Institute of Biochemistry, Vilnius, Lithuania). NQO1 was prepared from rat liver according to Prochaska [53]; its concentration was determined according to ε_460_ = 11.0 mM^−1^·cm^−1^. 2-(4-Chlorophenyl)sulfonyl-6-methoxy-3-nitropyridine (Tri-1), 6-nitro-1-benzothiophene 1,1-dioxide (Stattic), 2-[(4-methylphenyl)sulfonyl]-5-nitrofuran (NSC697923) and 2-nitro-5-(phenylsulfonyl)phenyl-4-chlorobenzoate (BTB06548) were obtained from Selleck Chemicals (Houston, TX, USA) and used as received. Rat cytosolic TrxR1 (110 U/mg), NADPH, and other reagents were obtained from Sigma-Aldrich (St. Louis, MO, USA) and used as received.

### 4.2. Enzymatic Assays and Chemical Reactions

The kinetic measurements were carried out spectrophotometrically using a Perkin Elmer Lambda 25 spectrophotometer (PerkinElmer, Waltham, MA, USA) in 0.1 M K-phosphate buffer (pH 7.0) containing 1 mM EDTA at 25 °C. The activities of P-450R and ADR/ADX were determined according to the reduction rate of 50 µM cytochrome *c* (∆ε_550_ = 20 mM^−1^·cm^−1^) at substrate concentrations indicated below. They were close to those reported previously [31,40]: 37 s^−1^ (P-450R, [NADPH] = 100 µM), 7.8 s^−1^ (ADR, [ADX] = 0.5 µM, [NADPH] = 50 µM), and 1750 s^−1^ (NQO1, [NADPH] = 150 µM, [menadione] = 10 µM). In this case, 0.01% Tween 20 and 0.25 mg/mL bovine serum albumin were added as NQO1 activators. The activity of HGR, determined according to the initial oxidation rate of 200 µM NADPH (∆ε_340_ = 6.2 mM^−1^·cm^−1^) in the presence of 1.0 mM GSSG, was equal to 170 s^−1^. The activity of TrxR2, determined according to the reduction rate of 2.0 mM DTNB (Δε_412_ = 27.2 mM^−1^·cm^−1^) in the presence of 200 µM NADPH, was close to that reported previously, 7.3 s^−1^ [37]. The activity of rat TrxR1, determined under the same conditions, was equal to 3.64 µmol DTNB reduced ·min^−1^ ·mg protein.

The initial rates of enzymatic NADPH-dependent ArNO_2_ reduction were determined according to ∆ε_340_ = 6.2 mM^−1^·cm^−1^ after the subtraction of intrinsic NADPH oxidase activities of enzymes, 0.06 s^−1^ (P-450R), 0.1 s^−1^ (NQO1), 0.13 s^−1^ (ADR + 0.5 µM ADX), 0.04 s^−1^ (HGR), and 0.02 s^−1^ (TrxR2). The stock solutions of oxidants were prepared in DMSO (dilution factor 100). The values of turnover rate, *k*_cat_, reflect the maximal number of moles of NADPH oxidized or oxidant reduced per mole of the enzyme active center per second at a saturating concentration of substrates. Additionally, *k*_cat_/*K*_m_ reflects the bimolecular rate constant (or catalytic efficiency constant) and corresponds to the inverse intercepts and slopes in Lineweaver–Burk coordinates, [E]/*v* vs. 1/[oxidant]. These rate constants were obtained by fitting the experimental data to the parabolic expression using SigmaPlot 2000 (version 11.0, Systal Software, San Jose, CA, USA). In some experiments, as noted in the main text, the NADPH regeneration system (10 mM glucose-6-phosphate and 3 µg/mL glucose-6-phosphate dehydrogenase) was used.

In the study of reversible inhibition of TrxR1 or HGR (t = 0), ArNO_2_ was introduced into the reaction mixture (concentrations of NADPH, DTNB, or GSSG were as mentioned above). The reaction was started by the introduction of the enzyme. The concentration of ArNO_2_ that decreased the reaction rate by 50% (IC_50_) was calculated in coordinates 1/*v* vs. [inhibitor]. Irreversible (time-dependent) inhibition of TrxR or HGR was tested by incubating the enzymes with 200 μM NADPH and a fixed concentration of ArNO_2_. After incubation, the reaction was started by the introduction of DTNB or GSSG. The pseudo-first-order inactivation rate constant (*k*_i_) was calculated in the coordinates ln *v* vs. incubation time.

The reactions of GSH (2.0–10.0 mM) with 50–100 µM nitroaromatic sulfones were studied spectrophotometrically at pH 7.0 and 25 °C. After the addition of GSH, the absorbance rise at 366 nm (NSC697923) or 367 nm (Tri-1), or its decrease at 315 nm (Stattic) or 280 nm (BTB06548), was monitored. The pseudo-first-order reaction rate constants were calculated according to a method by Guggenheim [54].

### 4.3. Cytotoxicity Assays

Murine hepatoma MH22a cells obtained from the Institute of Cytology of the Russian Academy of Sciences (St. Petersburg, Russia) were grown and maintained at 37 °C in DMEM medium supplemented with 10% fetal bovine serum, 100 U/mL penicillin, and 0.1 mg/mL streptomycin, as described in [31,40]. In the cytotoxicity experiments, 3.0 × 10^4^/mL cells were seeded on glass slides in 5 mL flasks, either in the presence or absence of compounds, and were grown for 24 h. In the absence of compounds, cells reached 40–50% confluence. Then, the slides were rinsed 3–4 times with phosphate-buffered saline and stained with Trypan blue. The cells adherent to the slides were counted under a light microscope. Typically, they did not accumulate Trypan blue, and their viability was 98.5–99.3%. Human colon adenocarcinoma cells HCT-116 obtained from ATCC (Manassas, VA, USA) were grown and maintained at 37 °C in 5% CO_2_ in RPMI 1640 DMEM medium, supplemented with 10% fetal bovine serum, 2 mM L-glutamine, and 0.05 mg/mL gentamycin. In the cytotoxicity experiments, 1.0 × 10^5^/mL cells were seeded in the absence or presence of compounds and were grown for 48 h. In the absence of compounds, cells reached 65–75% confluence. Their viability was determined by staining with crystal violet [55]. Stock solutions of compounds were prepared in DMSO. Its concentration in cultivation media did not exceed 0.2% and did not affect cell viability. The experiments were conducted in triplicate.

### 4.4. Studies of Thioredoxin Reductase and Glutathione Reductase Activity in Cell Lysates

For the enzymatic analysis, MH22a and HCT-116 cells were grown until confluence, detached by trypsinization, twice washed with PBS, and sonicated on ice in four cycles of 20 s. The homogenate was centrifuged at 14,000× *g* for 45 min, and the resulting supernatant with added 1.0 mM PMSF was used for enzymatic analysis. Protein concentration was determined according to the Bradford method. The activity measurements were performed in 0.1 M K-phosphate buffer (pH 7.0) containing 1 mM EDTA at 37 °C, with a final protein concentration of 80 µg·mL^−1^. The activity of TrxR was determined according to the reduction rate of 2.0 mM DTNB in the presence of 200 µM NADPH, as described in [56]. In this case, the activity is expressed as the difference between the reaction rates before and after the sample incubation with NADPH and 2.0 µM auranofin for 20 min. The initial rates were corrected for the nonenzymatic reduction rates of DTNB by the low m.w. thiols present in the sample. Typically, the treatment with auranofin suppressed the rate of DTNB reduction by ~95%. The activity of GR was determined according to the oxidation rate of 200 µM NADPH by 1.0 mM GSSG, monitored at 340 nm. Studying the effects of ArNO_2_ on the activity of TrxR and GR, the reaction rates were recorded as follows: (a) In the absence of compound; (b) Immediately after the addition of compound; (c) After the protein incubation in the presence of NADPH for 20 min; and (d) After the protein incubation in the presence of NADPH and compound for 20 min.

### 4.5. Statistical Analysis and Calculations

The statistical analysis was performed using Statistica (version 4.3, Statsoft, Toronto, ON, Canada). Octanol/water distribution coefficients at pH 7.0 (log *D*) were calculated using the Log *D* Predictor (https://chemaxon.com (accessed on 23 May 2022)).

## 5. Conclusions

The studies of enzymatic single-electron reduction in known or identified in this work nitroaromatic inhibitors of TrxR and the calculation of their *E*^1^_7(calc.)_ enabled us to quantitatively compare their cytotoxicity with that of model redox cycling ArNO_2_. This made it possible to determine whether their cytotoxicity could be linked to TrxR inhibition or other factors. We also identified NSC697923 as a novel, potent inhibitor of TrxR, which also inhibits HGR. Our data may be instrumental in the search for nitroaromatic TrxR inhibitors with enhanced cytotoxic/anticancer activity. Future studies may be directed at evaluating the role of reversible TrxR inhibition, possible synergistic effects of TrxR and GR inhibition, and the influence of NQO1 and cytochromes P-450 on the activity of inhibitors.

## Figures and Tables

**Table 1:** The bimolecular rate constants of reduction in nitroaromatics by NADPH:cytochrome P-450 reductase (P-450R), adrenodoxin reductase/adrenodoxin (ADR/ADX), and NAD(P)H:quinone oxidoreductase (NQO1) (0.1 M K-phosphate, pH 7.0, 25 °C), and their calculated *E*^1^_7_ values (*E*^1^_7(calc.)_).

Compound	*k*_cat_/*K*_m_ (M^−1^s^−1^)			*E*^1^_7(calc.)_ (V)
	P-450R	ADR/ADX	NQO1	
1-Chloro-2,4-dinitro- benzene	1.7 ± 0.1 × 10^5^	3.6 ± 0.2 × 10^5^	≤10^2^ (≤0.1) ^a^	−0.285
NSC697923	5.0 ± 0.4 × 10^4^	1.1 ± 0.1 × 10^6^	2.5 ± 0.4 × 10^3^ (≤0.25) ^a^	−0.287
Stattic	1.3 ± 0.1 × 10^5^	1.6 ± 0.1 × 10^5^	6.8 ± 0.8 × 10^2^ (0.15 ± 0.02) ^a^	−0.304
BTB06584	1.6 ± 0.1 × 10^4^	3.7 ± 0.2 × 10^5^	(≤0.07) ^b^	−0.326
Tri-1	1.7 ± 0.1 × 10^4^	3.7 ± 0.2 × 10^4^	2.1 ± 0.2 × 10^3^ (0.10 ± 0.02) ^a^	−0.365

^a^ The values of *k*_cat_ (s^−1^) are given in parentheses, ^b^ rate at the solubility limit of BTB06548, 70 µM.

**Table 2:** Concentrations of nitroaromatic compounds causing 50% MH22a cell death (cL_50_) and 50% growth inhibition of HCT-116 cells (GI_50_), their calculated single-electron reduction potentials (*E*^1^_7(calc.)_), and octanol/water partition coefficients at pH 7.0 (log *D*). The values of cL_50_ or GI_50_ predicted according to Equations (A2) or (A3) (Appendix B) are given in parentheses.

Compound	*E*^1^_7(calc.)_ (V)	log *D*	cL_50_ MH22a (µM)	GI_50_ HCT-116 (µM)
CDNB	−0.285	2.46	1.9 ± 0.1 (28.3)	5.0 ± 0.3 (40.2)
NSC697923	−0.287	2.70	1.0 ± 0.1 (25.1)	1.0 ± 0.1 (38.3)
Stattic	−0.304	1.12	1.8 ± 0.2 (100)	2.0 ± 0.2 (106)
BTB06584	−0.326	5.13	28.0 ± 4.0 (10.5)	80.0 ± 9.0 (36)
Tri-1	−0.365	3.29	6.6 ± 0.5 (77.6)	7.0 ± 0.7 (184)

**Table 3:** Modulation of cytotoxicity of nitroaromatic compounds in MH22a cells by antioxidants, dicoumarol, and inhibitors of cytochromes P-450, *n* = 3, *p*  <  0.05 *, *p*  <  0.02 **, *p*  <  0.005 *** with respect to cell viability in the absence of additions. The indicated concentrations of the above compounds did not affect cell viability by more than ±2%. The cell viability data are adjusted to these changes.

Compound	Cell Viability (%)						
	Additions:						
	None	DPPD (2.0 µM)	Desferri- Oxamine (1.0 mM)	Dicoumarol (20 µM)	α-Naphtho- Flavone (5.0 µM)	Isoniazid (1.0 mM)	Miconazole (5.0 µM)
1-Chloro-2,4-dinitrobenzene (2.0 µM)	49.3 ± 4.2	77.3 ± 5.6 ***	67.0 ± 4.6 **	69.5 ± 4.8 **	63.3 ± 3.8 *	64.9 ± 4.8 *	74.9 ± 7.7 **
NSC697923 (1.0 µM)	45.1 ± 3.5	55.7 ± 5.2	61.7 ± 4.4 **	63.1 ± 5.0 **	69.0 ± 6.4 **	71.0 ± 5.5 ***	71.0 ± 5.3 ***
Stattic (2.0 µM)	50.3 ± 5.5	79.1 ± 6.6 **	75.0 ± 3.1 **	76.7 ± 10 *	38.6 ± 4.8 *	32.6 ± 2.8 ***	74.5 ± 10.7 *
Tri-1 (6.0 µM)	49.9 ± 4.5	67.7 ± 4.6 **	64.7 ± 4.3 *	78.3 ± 4.8 ***	70.5 ± 4.6 **	77.0 ± 4.8 ***	68.6 ± 3.3 **
Tetryl (10.0 µM)	45.5 ± 4.2	78.4 ± 5.8 ***	79.9 ± 5.0 ***	28.2 ± 2.8 ***	55.4 ± 5.0	54.4 ± 3.6	24.2 ± 1.1 ***

**Table 4:** Concentrations of compounds cause a 50% decrease in the initial rate of TrxR1- or HGR-catalyzed reactions (IC_50_) and the rate constants of enzyme inactivation (*k*_i_) at pH 7.0 and 25 °C. The concentrations of compounds used in inactivation experiments are given in parentheses.

Compound	Enzyme			
	Rat TrxR1		HGR	
	IC_50_ (µM)	*k*_i_ (min^−1^)	IC_50_ (µM)	*k*_i_ (min^−1^)
1-Chloro-2,4-dinitrobenzene	25 ± 5.0	0.10 ± 0.01 (10 µM)	40 ± 6.0	≤0.005 (10 µM)
NSC697923	1.1 ± 0.1	0.12 ± 0.02 (1.0 µM)	0.5 ± 0.1	0.02 ± 0.003 (1.0 µM)
Stattic	15 ± 2.0	0.14 ± 0.02 (10 µM)	14.0 ± 1.0	0.02 ± 0.003 (10 µM)
Tri-1	2.0 ± 0.3	0.12 ± 0.01 (1.0 µM)	32.0 ± 5.0	≤0.005 (10 µM)
Tetryl	10 ± 1.3	0.04 ± 0.01 (1.0 µM) 0.30 ± 0.04 (10 µM)	14 ± 2.0	≤0.002 (10 µM)

**Table 5:** The summary of the data on the TrxR and GR inhibition during the incubation of the cells with the irreversible inhibitors of TrxR.

Compound	Cells	Compound Concentration, Incubation Time	Residual Activity (%)	
			TrxR	GR
Tri-1	HCT-116 [23]	3.3 µM, 3 h	20	n.d.
	BF16-F10 [44]	2.0 µM, 3 h	50	n.d.
		20 µM, 3 h	10	n.d.
Stattic	A549 [22]	10 µM, 4 h	25	n.d.
	Various human cervical carcinoma cells [24]	20 µM, 0.5 h	n.d.	30–50
1-Chloro-2,4-dinitrobenzene	A549 [21]	50 µM, 4 h	30	80
	HeLa [21]	50 µM, 4 h	30	100
Tetryl	A549 [21]	50 µM, 4 h	80	100
	HeLa [21]	50 µM, 4 h	75	100

## Data Availability

The data could be available from the corresponding author upon reasonable request.

## Appendix A

Table A1 contains the previously determined logarithms of geometric averages of *k*_cat_/*K*_m_ of model ArNO_2_ in P-450R- and ADR/ADX-catalyzed reactions (log *k*_cat_/*K*_m_ (avge) = 0.5 log *k*_cat_/*K*_m_ (P-450R) + 0.5 log *k*_cat_/*K*_m_ (ADR/ADX)) [31,40], supplemented by the repeated studies of some previously studied compounds. For quantitative analysis, when available, data obtained in the current work were used.

**Table A1:** Single-electron reduction midpoint potentials of nitroaromatic compounds (*E*^1^_7_), their bimolecular reduction rate constants (*k*_cat_/*K*_m_) by NADPH:cytochrome P-450 reductase (P-450R) and adrenodoxin reductase/adrenodoxin (ADR/ADX), and their geometric averages (log *k*_cat_/*K*_m_ (avge)). Data were taken from [29,38]. The values obtained in the current work are given in parentheses.

No.	Compound	E^1^_7_ (V) ^a^	*k*_cat_/*K*_m_ (M^−1^s^−1^)		log *k*_cat_/*K*_m_ (avge)
			P-450R	ADR/ADX	
1	Nitrobenzene	−0.485	6.8 ± 0.8 × 10^2^	3.4 ± 0.2 × 10^3^	3.18
2	4-Nitrobenzoic acid	−0.425	2.3 ± 0.2 × 10^3^ (2.9 ± 0.2 × 10^3^)	2.0 ± 0.2 × 10^3^ (2.3 ± 0.2 × 10^3^)	3.33 (3.41)
3	2-Nitrothiophene	−0.390	1.4 ± 0.1 × 10^4^	4.2 ± 0.3 × 10^4^	4.38
4	4-Nitroacetophenone	−0.355	1.7 ± 0.2 × 10^4^	3.2 ± 0.3 × 10^4^	4.37
5	1,3-Dinitrobenzene	−0.345	4.9 ± 0.2 × 10^4^	5.2 ± 0.4 × 10^4^	4.70
6	4-Nitrobenzaldehyde	−0.325	3.3 ± 0.2 × 10^4^	1.7 ± 0.3 × 10^5^	4.87
7	2-Nitrothiophene-5-carboxymorpholide	−0.305	1.4 ± 0.2 × 10^5^	4.1 ± 0.6 × 10^5^	5.38
8	1,2-Dinitrobenzene	−0.287	1.6 ± 0.1 × 10^5^	1.8 ± 0.2 × 10^5^	5.23
9	2-Nitrothiophene-5-aldoxime	−0.280	2.2 ± 0.2 × 10^6^ (1.8 ± 0.2 × 10^6^)	5.4 ± 0.5 × 10^5^ (4.2 ± 0.4 × 10^5^)	6.04 (5.94)
10	2-Nitrothiophene-5-aldehyde	−0.260	2.8 ± 0.1 × 10^5^	4.0 ± 0.5 × 10^5^	5.52
11	Nitrofurantoin	−0.255	9.1 ± 1.4 × 10^4^	1.0 ± 0.2 × 10^6^	5.48
12	Nifuroxime	−0.255	1.1 ± 0.1 × 10^5^	1.0 ± 0.1 × 10^6^	5.52
13	1,4-Dinitrobenzene	−0.255	1.2 ± 0.1 × 10^6^	2.0 ± 0.2 × 10^6^	6.19
14	2,4,6-Trinitrotoluene	−0.253	1.0 ± 0.1 × 10^5^ (1.4 ± 0.1 × 10^5^)	7.3 ± 0.2 × 10^5^ (7.0 ± 0.2 × 10^5^)	5.43 (5.50)
15	Tetryl	−0.191	5.9 ± 0.2 × 10^6^	8.9 ± 1.0 × 10^5^	6.36

^a^ From Refs. [13,34,57].

The relationship between log *k*_cat_/*K*_m_ (avge) and *E*^1^_7_ of nitroaromatics is described by Equation (A1):

log *k*_cat_/*K*_m_ (avge) = (8.64 ± 0.30) + (11.47 ± 0.94) *E*^1^_7_, *r*^2^ = 0.9209, F(1,13) = 150.11. (A1)

## Appendix B

We have shown that the cytotoxicity of model nitroaromatic compounds without alkylating groups towards MH22a and HCT-116 cells increases with their *E*^1^_7_ and log *D* values [31,40]. Table A2 presents our previous data, supplemented by studies of some new compounds or repeated studies of some previously studied compounds. For quantitative analysis, data obtained in the current work were used when available.

**Table A2:** Single-electron reduction midpoint potentials of nitroaromatic compounds (*E*^1^_7_), their octanol/water distribution coefficients at pH 7.0 (log *D*), and their concentrations causing 50% death of MH22a cells (cL_50_) or inhibiting the growth of HCT-116 cells by 50%.

No.	Compound	*E*^1^_7_ (V)	log *D*	MH22a cL_50_ (μM)	HCT-116 GI_50_ (μM)
1	Nitrobenzene	−0.485	1.91	1800 ± 200	≥5000
2	4-Nitrobenzoic acid	−0.425	−1.66	≥6000	≥6000
3	2-Nitrothiophene	−0.390	1.86	341 ± 42	600 ± 70 ^a^
4	4-Nitroacetophenone	−0.355	1.47	239 ± 19	400 ± 80
5	1,3-Dinitrobenzene	−0.345	1.85	130 ± 14	350 ± 20
6	4-Nitrobenzaldehyde	−0.325	1.63	200 ± 15	50.0 ± 6.0
7	2-Nitrothiophene-5-carboxymorpholide	−0.305	1.07	82.0 ± 12	n.d.
8	1,2-Dinitrobenzene	−0.287	1.85	25.4 ± 3.0	60.0 ± 10.0
9	2-Nitrothiophene-5-aldoxime	−0.280	1.7	145 ± 30 118 ± 20 ^a^	20.0 ± 5.0
10	2-Nitrothiophene-5-aldehyde	−0.260	1.26	42.0 ± 5.0	20.0 ± 3.0 ^a^
11	Nitrofurantoin	−0.255	−0.25	387 ± 25 320 ± 25 ^a^	60.0 ± 10.0
12	Nifuroxime	−0.255	−0.34	40.5 ± 5.0	70.0 ± 10.0
13	1,4-Dinitrobenzene	−0.255	1.85	12.0 ± 1.5	40.0 ± 7.0
14	2,4,6-Trinitrotoluene	−0.253	2.31	17.4 ± 2.0	40.0 ± 8.0
15	Tetryl	−0.191	1.38	7.0 ± 1.0	8.0 ± 1.5

^a^ Data from this work.

The relationship between log cL_50_ towards MH22a cells and *E*^1^_7_ of nitroaromatic compounds is described by Equation (A2):

log cL_50_ = −(0.27 ± 0.32) − (8.55 ± 0.94) *E*^1^_7_ − (0.29 ± 0.07) log *D*, *r*^2^ = 0.9011, F(2,12) = 54.67. (A2)

The relationship between log GI_50_ towards MH22a cells and *E*^1^_7_ of nitroaromatic compounds is described by Equation (A3):

log GI_50_ = −(0.83 ± 0.28) − (10.03 ± 0.82) *E*^1^_7_ − (0.17 ± 0.06) log *D*, *r*^2^ = 0.9372, F(2,11) = 82.10. (A3)
